# Factors that influence the use of direct access to allied health professionals in the Netherlands

**DOI:** 10.1017/S1463423625100467

**Published:** 2025-09-23

**Authors:** Laura J. Damen, Willemijn M. Meijer, Lilian H.D. Van Tuyl, Bart J. Knottnerus, Judith D. De Jong

**Affiliations:** 1 Nivel, the Netherlands Institute for Health Services Research, Utrecht, the Netherlands; 2 CAPHRI, Maastricht University, PO Box 616, 6200 MD Maastricht, Maastricht, the Netherlands

**Keywords:** Allied health professionals, direct access, healthcare system, primary healthcare

## Abstract

**Introduction:**

Healthcare systems worldwide are under pressure due to increasing demand and rising costs. Simultaneously, there is a shortage of healthcare workers. This is leading to increased pressure on primary care, especially in countries where general practitioners (GPs) perform a gatekeeping function. One way to alleviate this pressure on GPs, and to reduce healthcare costs, is to introduce or expand, direct accessibility to allied health professionals. This study investigated the factors associated with this direct accessibility in the Netherlands.

**Method:**

We used data from electronic health records of physiotherapists, speech therapists, and dietitians, drawn from the 2022 Dutch Nivel Primary Care Database (Nivel’s PCD). The data included information ranging from 15,470 to 776,690 patients, and for 62 to 593 practices, depending on the particular paramedic discipline. Multilevel logistic regressions were employed to identify patient and practice characteristics associated with direct access.

**Results:**

Patient characteristics significantly associated with direct access included younger age, higher socioeconomic status, and diagnosis. The patient’s sex was also identified as a factor associated with the use of direct access in physiotherapy and dietetics, but not in speech therapy. Moreover, we observed significant variation between practices. We found that the dominant health insurer in an area was sometimes associated with direct access, as well as the number of therapists working in a practice.

**Conclusion:**

We observed significant associations between patient and practice characteristics and the direct access to allied health professionals in primary care. These findings suggest that the use of direct access to allied health professionals could be increased in order to enhance healthcare efficiency and thereby relieve pressure on GP care.

## Introduction

Healthcare systems around the world are facing pressure due to the increasing demand for, and costs of, healthcare (Rechel *et al.*, 2009, Rudnicka *et al.*, 2020, Organisation for Economic Co-operation and Development, 2021a, Organisation for Economic Co-operation and Development, 2021b). Simultaneously, there is a shortage of workforce (Liu *et al.*, 2017, Organisation for Economic Co-operation and Development, 2021b, Boniol *et al.*, 2022). This is leading to an increased pressure on primary care, in particular in countries where general practitioners (GPs) have a gatekeeping function. The gatekeeping role of GPs in these systems means that they are the first point of contact for most requests for healthcare, thus increasing their workload significantly. The high demand results in reduced quality of care, longer waiting times, and higher rates of burnout among primary care providers (Gunja *et al.*, 2022, Beech *et al.*, 2023).

One approach to enhance healthcare efficiency, thus relieving pressure on GP care (Foster *et al.*, 2012, Goodwin and Hendrick, 2016), and reducing healthcare costs (Mitchell and de Lissovoy, 1997, Denninger *et al.*, 2018, Gallotti *et al.*, 2023), is to introduce, or expand, direct access to allied health professionals. Such direct access is already available in various countries such as the Netherlands, the United Kingdom, the United States, and Denmark (Foster *et al.*, 2012). It allows patients to seek care from allied health professionals, such as physiotherapists, without needing a referral from a GP or another healthcare professional (HCP). Research has shown that patients who use direct access often achieve similar or even better clinical outcomes compared to those whose first contact is with a GP or other HCPs (Overman *et al.*, 1988, Holdsworth *et al.*, 2006, Brooks *et al.*, 2008, Ludvigsson and Enthoven, 2012, Goodwin and Hendrick, 2016, Bishop *et al.*, 2017, Bornhöft *et al.*, 2019). During an initial appointment without a referral, the allied health professional evaluates whether treatment is appropriate and safe. They can check for any so called *red flags*, which are symptoms or conditions outside their scope of practice. In such cases, the allied health professional refers the patient to their GP. Despite this possibility for direct access, in a recent study, Dutch GPs reported that many patients still unnecessarily seek referrals from them (Damen *et al.*, 2024).

In the Netherlands, reports to the annual Nivel Primary Care Database (Nivel PCD) showed that 70.5% of the patients sought treatment from physiotherapists without a referral in 2022 (Veldkamp *et al.*, 2023). The rate of self-referral is notably lower for speech therapy, with only 19.5% of patients choosing this route in 2022 (Meijer, 2023). It is even less common for dietitians, accounting for only 15.2% of patients in 2022 (Zinger and Meijer, 2023). These findings suggest that there may be a potential for greater use of direct access. To explore this potential, it is important to examine patient and practice characteristics that may explain the use of direct access.

We aimed in this study to identify factors associated with the use of direct access to allied health professionals in the Netherlands. This investigation is important for informing policy decisions geared towards efficiency in providing care. Increasing the use of direct access may help avoid unnecessary pressure on general practices as it allows for targeted interventions tailored to specific patient groups and practices. Previous studies comparing self-referrals with standard doctors’ referrals have focused predominantly on just physiotherapy (Overman *et al.*, 1988, Holdsworth *et al.*, 2006, Brooks *et al.*, 2008, Ludvigsson and Enthoven, 2012, Goodwin and Hendrick, 2016, Bishop *et al.*, 2017, Bornhöft *et al.*, 2019). Furthermore, while differences in the use of direct access among patients have been explored in previous research (Scheele *et al.*, 2014, Holdsworth *et al.*, 2006, Veldkamp *et al.*, 2022), there are few studies investigating variations between practices. This study adressed the following research question: *What factors influence the use of direct access to allied health professionals in the Netherlands?* The following sub-questions were answered: (1) *What patient characteristics contribute to differences in the use of direct access between patients*?*; (2) What practice characteristics contribute to differences in the use of direct access between practices?*

Information from Nivel’s PCD, which included data from physiotherapists, exercise therapists, dietitians, and speech therapists about the care they provide are used to answer these questions. We should note that internationally, exercise therapy is often incorporated, into physiotherapy, but in the Netherlands, it is a recognized form of treatment aimed at promoting healthy movement (Veldkamp, 2023a). However, the two professions share many similarities, including compensation structures. Therefore, we combined, for this study, data from both physiotherapy and exercise therapy and referred to this combination as physiotherapy.

## Methods

### Description data

We used electronic health record data from Dutch physiotherapists, speech therapists, and dietitians, which were collected within Nivel’s PCD for 2022. Nivel’s PCD gathers longitudinal data systematically thus encompassing patient characteristics, treatment registrations, patterns in the use of care, health problem diagnoses, and treatment evaluations (Nivel Research Communication Center, 2024). The practices participating were distributed across the Netherlands and were representative of the country’s practices in terms of the sex and age of allied health professionals, practice location, and practice types (Veldkamp, 2023b, Nivel Research Communication Center, 2024, Meijer, 2024a, Meijer, 2024b, Veldkamp, 2024). In 2022, the physiotherapy data consisted of information on 604 practices and 783,194 patients. The speech therapy data consisted of 192 practices and 32,140 patients. And the dietetics dataset included 87 practices and 24,202 patients (Meijer, 2023, Veldkamp *et al.*, 2023, Zinger and Meijer, 2023).

### Sample

The study sample comprised incident treatment episodes in physiotherapy, speech therapy, and dietetics practices participating in the Nivel PCD during 2022. These were defined as episodes with a first contact in that year. We included only the first treatment session in instances where patients initiated multiple treatment episodes within a single year. This was because the number of patients with multiple incidents was too small for analysis on an individual level. Additionally, practices that exclusively relied on referrals were excluded from the study, as patients in these settings did not have the option of choosing between direct access and referral. Only practices that used direct access at least once were included resulting in a study sample of 593 physiotherapy practices (*n* patients = 776.882), 110 speech therapy practices (*n* patients = 21.201), and 62 dietitian practices (*n* patients = 15.470). The practices which were excluded as they relied solely on referrals, were examined further in order to assess potential differences from the practices we included (see additional analyses).

### Data sources

The Nivel PCD includes information from routine electronic health records from, among others, allied health practices in primary care. This is based on regular routine administration supplemented with data from reporting guidelines (Veldkamp, 2023b, Meijer, 2024a, Meijer, 2024b, Veldkamp, 2024). The data contained the following patient and practice characteristics: the sex and age of the patient, the primary health problem or diagnosis, and the number of therapists in a practice. For physiotherapy, information about the number of incident treatment episodes in 2022 per patient was included as well. Information about whether patients consulted allied health professionals via direct access or through referral from a doctor, such a GP or specialist, was derived from a defined circumstance. This was that the paramedic explicitly indicated that a patient arrived via direct access. In cases where such explicit indication was absent, direct access was inferred based on performance codes, used specifically for the reimbursement of costs.

Further variables were incorporated by integrating additional datasets, for example by linking patients’ postal codes to data from Statistics Netherlands thus enabling us to include socioeconomic status (SES) based on their postal codes as a variable (Centraal Bureau voor de statistiek (CBS), 2024a). The SES score derived from postal codes and based on affluence, educational attainment, and participation in the labour market, is publicly available and reflects the average SES of the neighbourhood. This score indicates how much the SES of a neighbourhood deviates from the national average, which is set at zero (Centraal Bureau voor de statistiek (CBS), 2024a). An SES based on postal codes was assigned to all patients within our data living in that location. As patients’ postal codes for physiotherapy were not available, this SES could only be included in the datasets of dietitians and speech therapists.

Similarly, the practice’s postal code from the Nivel PCD dataset was combined with urbanization data from Statistics Netherlands allowing us to classify the practice locations. We were able to allocate them to three degrees of urban living: strongly or very strongly, moderately, and not at all (Centraal Bureau voor de statistiek (CBS), 2024b).

Additionally, data on the dominant health insurer in each area were included. This refers to the health insurance company with the largest market share or majority of enrollees in a particular region. This information is publicly available (RIVM, 2023) and was linked to the Nivel PCD by also using the practices’ postal codes.

### Study setting and definitions

Age groups were created to address the non-linear relationship between direct access and age. These groups included children aged 0 to 12 years, followed by adolescents aged 13 to 17 years. This differentiation was made because children older than 12 years may legally participate in decisions about their medical care. Prior to that age parents or legal representatives may make decisions for the children (The Royal Dutch Medical Association (RDMA), 2021). Additional age groups were defined as 18–40, 41–64, and 65 years or older. The variable SES based on postal codes in the datasets of dietetics and speech therapy was tested for linearity and this was confirmed. Health insurers in the study are labelled using the letters of the alphabet from A to G, with their actual names intentionally withheld.

Patients treated by allied health professionals could present many different symptoms, which leads to a wide variety of diagnoses in the registries. We were not interested in specific diagnoses, but rather in the association of the diagnosis with the decision of whether to consult the allied health professional through direct access. We, therefore, clustered the diagnoses per discipline. This clustering varied among the three disciplines. Within physiotherapy and dietetics, there are certain patient groups who are more likely to access care via referral and for whom it is also desirable that they do so. Among the remaining groups, it is of interest to explore whether there is still potential to increase the use of direct access. The classification was made on this basis. In the case of speech therapy, however, such a distinction could not be made, and the classification is based solely on diagnostic groups.

For physiotherapy, the frequency and extent to which a treatment is reimbursed is complex and depends upon several factors. Most treatments are not included in the basic health insurance package, but it is possible to have an additional insurance that covers a predetermined number of treatments and some treatments have to be paid for out of pocket (Vektis, 2024b, Kennis- en exploitatiecentrum Officiële Overheidspublicaties, 2024). In many cases, a referral from a doctor is required for treatment to be reimbursed by basic health insurance. It is therefore likely that patients with a diagnose that is reimbursed from the basic insurance access physiotherapists with a referral more often than patients with a diagnose that is not in the basic insurance. Consequently, we have grouped diagnoses into those that are reimbursed by basic health insurance and those that are not.

For speech therapy, we distinguished between four diagnoses: language disorders, articulation disorders, disorders in sensory motor skills of the mouth and ‘other’. The other category included hearing impairment, voice impairment, reading and writing impairment, and COVID-19 infection.

Dietitians could record multiple diagnoses per patient, with a maximum of four (Vektis, 2024a). We grouped the diagnoses into three categories. First of all, we distinguished medical conditions, with or without other dietitian diagnoses, as these patients are, by definition, treated by physicians for this condition which then increases the likelihood of a referral to the dietitian. Medical conditions included: hypertension/heart disease, infectious diseases, pulmonary diseases, gastrointestinal liver diseases, metabolic diseases, neurological diseases, renal diseases, oncology, mental/behavioural disorders, rheumatic diseases, COVID-19 infection, surgery, and eating disorders. For the remaining patients, we distinguished being overweight as a condition, regardless of whether a patient also had another condition. The last category, ‘other’, encompassed disorders related to nutrition such as food allergies or food intolerance, general symptoms such as swallowing complaints or chewing and dental problems, and artificial nutrients.

We generated interaction terms between each age group and each category of diagnosis in order to examine whether the relationship between diagnosis and the outcome variable varies across different age groups.

We constructed variables at the level of each practice using patient data. These included the average age of the patient, their SES derived from their post codes, and the most common category of diagnosis within each practice. We expected that practices with older patient populations, for example, might treat a higher proportion of chronic illnesses. These patients are more likely to visit a GP first, either due to the chronic nature of their conditions or because a referral is required. This could have an impact upon the percentage of direct access cases within a practice.

For physiotherapy, we defined the average diagnosis as the percentage of reimbursed diagnoses within a practice. This resulted in a numerical variable. For dietitians, we classified practices based on their treatment of medical conditions. Those that treated medical conditions in half or more of cases were deemed primarily medical. Those with fewer were classified as non-medical. This created a categorical variable. For speech therapy, we were unable to compute an average diagnosis.

### Statistical analysis

A multilevel mixed-effects logistic regression was conducted to identify factors associated with the use of direct access. This was the dependent variable. We applied four different models, checking after each step whether the new model provided a significantly better fit. Only the final model is presented in the results. We started with a null model (Model 0) to assess whether there were any differences at the practice level. In Model 1, patient characteristics were added, including age, sex, primary health problem or diagnosis, SES, and the number of times a patient went to physiotherapy for different treatment episodes in the year 2022. Model 2 introduced interaction terms between diagnosis and age. Finally, Model 3 incorporated characteristics at the practice level. These comprised: the number of therapists per practice, the degree of urbanization of the practice location, the average patient age within the practice, the average SES derived from postal codes of patients in the practice, the average diagnosis of patients in the practice, and the dominant health insurer in the practice location. The analyses were conducted using Stata (StataCorp. 2019. Stata Statistical Software: Release 16. College Station, TX: StataCorp LLC.).

Some variables had missing values, though these were mostly minimal. They ranged between 0.02% and 2.07% of the total sample, consistent across all disciplines. We assessed the missing values for non-random patterns but found none, allowing for listwise deletion.

The study was reported in accordance with the RECORD guidelines for studies using routinely collected health data (Benchimol *et al.*, 2015).

### Additional analyses

We conducted two additional analyses. Firstly, there were a large number of practices, especially in speech therapy (*n* = 82) and dietetics (*n* = 24), where the average rate of direct access was 0%. Therefore, we compared the population and practice characteristics of these practices with those of the entire dataset and with practices having the highest average of direct access. Differences were observed between practices where the majority of patients accessed allied health professionals directly and those where all patients were referred. The primary differences were related to age and diagnosis. In dietetics and speech therapy practices that relied solely on referrals, the patient population tended to be older. However, physiotherapy practices that only treated patients referred to them, saw more children aged 0-12. Additionally, dietitians’ practices working soley with referred patients had a higher prevalence of medical diagnoses compared to those that primarily used direct access.

We conducted a sensitivity analysis, using a separate logistic multilevel model that excluded children (patients aged 0–17) to examine whether their inclusion might lead to outliers or unusual patterns in the results. We made this adjustment because children represent a unique group for several reasons. Firstly, they may visit allied health professionals for different complaints or diagnoses than adults. Secondly, the decision to pursue direct access or referral is typically made by their parents. Thirdly, many treatments for children are reimbursed through basic insurance, which differs with adults. The results of these analyses were not significantly different from those of the total dataset. The most notable differences were observed in speech therapy. However, since children make up more than 75% of this dataset – and are in fact the primary target group for speech therapy – removing them resulted in a fundamentally different and non-comparable population. Given that the inclusion of children more accurately reflects the real-world patient population in speech therapy, and that the exclusion of children had little impact on the results for physiotherapy and dietetics, we chose to proceed with the analysis including children.

## Results

The characteristics of the patients and practices per discipline are shown in Tables 1-3. The majority of patients of physiotherapy, and dietetics were women, unlike patients consulting a speech therapist, where over half of the patients were men. The age of patients also differs per discipline with speech therapists being consulted predominantly by children compared to physiotherapists and dietitians, who were consulted mostly by adults. Consequently, the mean age per practice among dietitians and physiotherapists was around 50 years, whereas in speech therapy it was 16 years. The average SES derived from postal codes of patients visiting dietitians and speech therapists matched the national average for the Netherlands (Centraal Bureau voor de statistiek (CBS), 2024a). Most practices in our datasets were located in highly urban areas, comprising around 50% of all practices.

**Table 1.** Characteristics physiotherapy ^A^

	Mean/ proportion	SD	Min	Max
Direct access				
• Referral	30.2%			
• Direct access	69.8%			
**Patient characteristics**				
Sex				
• Female	58.7%			
• Male	41.3%			
Age				
• 0–12	3.8%			
• 13–17	4.2%			
• 18–40	27.5%			
• 41–64	38.8%			
• ≥65	25.8%			
Diagnosis				
• Reimbursed	19.9%			
• Not reimbursed	80.1%			
Number of times physiotherapy^B^	1.1	0.303	1	7
**Practice characteristics**				
How urban^C^				
• Highly	51.1%			
• Moderate	19.8%			
• Rural	29.2%			
Dominant health insurer in the area of the practice				
• A	15.1%			
• B	49.2%			
• D	0.7%			
• C	1.3%			
• E	19.1%			
• F	11.0%			
• G	3.7%			
Average age of patients in the practice	49.1	6.047	5.8	81.2
Average percentage of diagnosis for which treatments are reimbursed within a practice^D^	0.2	0.096	0	1
Number of therapists per practice	25.6	27.492	1	137

Data source: Nivel Primary Care Database (2022).

N patients = 776.882; N practices = 593.

Notes:

A ) Physiotherapy = 98,09%; exercise therapy = 1,91%.

B ) Number of times physiotherapy = the number of times a patient went to visit the physiotherapist in 2022.

C ) The level of urbanization is determined by the average ambient address density, categorized as follows: (1) Highly urbanized area: with an average ambient address density of 1,500 or more addresses per square kilometre. (2) Moderately urbanized area: characterized by an average ambient address density ranging from 1,000 to 1,500 addresses per square kilometre. (3) Less urban/rural area: exhibiting an average ambient address density of 999 or fewer addresses per square kilometre (39).

D ) Average percentage of diagnosis for which treatments are reimbursed within a practice: we calculated the average diagnosis by assessing the percentage of reimbursed diagnoses within a practice.

**Table 2.** Characteristics speech therapy

	Mean/ proportion	SD	Min	Max
Direct access				
• Referral	73.6%			
• Direct access	26.4%			
**Patient characteristics**				
Sex				
• Female	45.3%			
• Male	54.7%			
Age				
• 0–12	74.5%			
• 13–17	4.7%			
• 18–40	5.9%			
• 41–64	7.4%			
• ≥65	7.5%			
SES^A^	0.0	0.250	−0.8	0.8
Diagnosis				
• Language disorder	37.6%			
• Articulation disorder	20.4%			
• abnormality sensory motor skills mouth	23.2%			
• Other speech therapy^B^	18.8%			
**Practice characteristics**				
How urban^C^				
• Highly	64.5%			
• Moderate	20.0%			
• Rural	15.5%			
Dominant health insurer in the area of the practice				
• A	17.9%			
• B	33.4%			
• D	0.5%			
• C	–			
• E	18.5%			
• F	23.9%			
• G	5.8%			
Average age of patients in the practice	16.1	8.438	2.9	50.7
Average SES of patients in the practice^A^	0.0	0.149	−0.4	0.3
Number of therapists per practice	10.9	8.975	1	34

Data source: Nivel Primary Care Database (2022).

*N* patients = 21,201; *N* practices = 110.

Notes:

A ) SES= socioeconomic status, which is based on affluence, educational attainment, and labour market participation, is publicly available and reflects the average SES of the neighbourhood (32).

B ) Other included hearing impairment, voice impairment, reading and writing impairment, and COVID-19 infection.

C ) The level of urbanization is determined by the average ambient address density, categorized as follows: (1) Highly urbanized area: with an average ambient address density of 1,500 or more addresses per square kilometre. (2) Moderately urbanized area: characterized by an average ambient address density ranging from 1,000 to 1,500 addresses per square kilometre. (3) Less urban/rural area: exhibiting an average ambient address density of 999 or fewer addresses per square kilometre (39).

**Table 3.** Characteristics dietitian

	Mean/ proportion	SD	Min	Max
Direct access				
• Referral	77.0%			
• Direct access	23.0%			
**Patient characteristics**				
Sex				
• Female	65.4%			
• Male	34.6%			
Age				
• 0–12	7.9%			
• 13–17	3.3%			
• 18–40	23.2%			
• 41–64	32.9%			
• ≥65	32.7%			
SES^A^	0.0	0.237	−0.8	0.8
Diagnosis				
• Medical	46.2%			
• Overweight	26.9%			
• Other^B^	26.9%			
**Practice characteristics**				
How urban^C^				
• Highly	54.4%			
• Moderate	14.5%			
• Rural	31.0%			
Dominant health insurer in the area of the practice				
• A	17.6%			
• B	23.4%			
• D	0.0%			
• C	0.7%			
• E	36.6%			
• F	20.4%			
• G	1.2%			
Average age of patients in the practice	50.6	7.762	17.5	62.3
Average SES of patients in the practice^A^	0.0	0.106	−0.2	0.3
Mean practice diagnoses^D^				
• 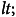 50% medical diagnoses within a practice	60.4%			
• 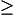 50% medical diagnoses within a practice	39.6%			
Number of therapists per practice	5.0	5.758	1	23

Data source: Nivel Primary Care Database (2022).

N patients=15,470; N practices = 62.

Notes:

A ) SES= socioeconomic status, which is based on affluence, educational attainment, and labour market participation, is publicly available and reflects the average SES of the neighbourhood (32).

B ) Other encompassed disorders related to nutrition such as food allergies or food intolerance, general symptoms such as swallowing complaints or chewing and dental problems, and artificial nutrients.

C ) The level of urbanization is determined by the average ambient address density, categorized as follows: (1) Highly urbanized area: with an average ambient address density of 1,500 or more addresses per square kilometre. (2) Moderately urbanized area: characterized by an average ambient address density ranging from 1,000 to 1,500 addresses per square kilometre. (3) Less urban/rural area: exhibiting an average ambient address density of 999 or fewer addresses per square kilometre (39).

D ) Mean practice diagnoses: we classified practices based on their treatment of medical conditions: those that treated medical conditions in 50% or more of cases were categorized as primarily medical, while those with less than 50% were classified as non-medical.

### Direct access or referral

**Figure 1.**
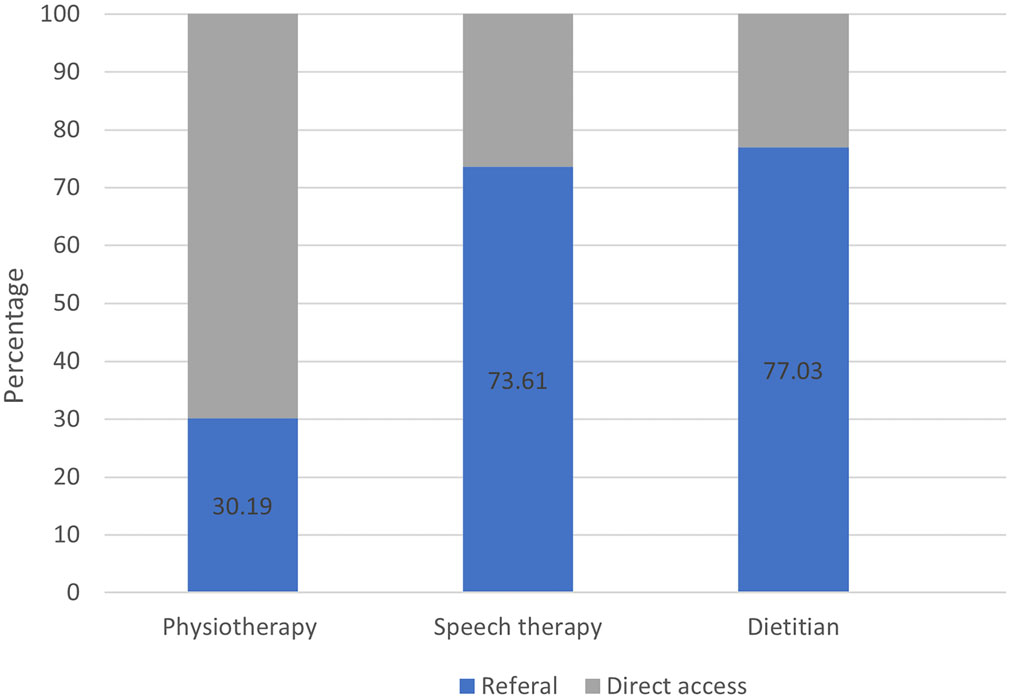
Percentage of patients consulting an allied healthcare provider via direct access (2022). Physiotherapy *n* = 776,686; speech therapy *n* = 21, 201; dietitian *n* = 15,470. *Note:* The percentage of direct access to speech therapy and dietitian is lower in reality than in the dataset. This discrepancy arose because practices that did not use direct access at all (0%) were removed from the dataset, as explained in the method section.

Figure 1 illustrates the distribution of episodes of care from patients who accessed allied health professionals either through direct access or referral, categorized by discipline. In physiotherapy, the majority of patients (69.8%) accessed care through direct access. Conversely, in speech therapy and among dietitians, the majority of patients (between 70% and 80%) were referred.

### Factors associated with direct access

Several factors were associated with the use of direct access. We will discuss them per discipline.

#### Physiotherapy

The patient characteristics associated with direct access to physiotherapy were sex, age, and whether the diagnosis is reimbursed (Table 4). Men were more likely to use direct access than women. Among age groups, the age group most likely to use direct access was 18–40 compared to the group aged 41–64. Furthermore, individuals aged 41–64 were more likely to use direct access than those aged 0–12, 13–17, or 65 and older. Patients with diagnoses for which treatments are reimbursed were less likely to use direct access compared to those with diagnoses for which treatments were not reimbursed. Additionally, the relationship between diagnosis and direct access varied across age groups. Specifically, the percentage of children (aged 0–17) using direct access increased when the treatments for their diagnosis were not reimbursed, compared to adults aged 41–64 with diagnoses for which treatments were not reimbursed.

**Table 4.** Physiotherapy^A^: patient and practice characteristics associated with the use of direct access

	Coef	*P* value	SE	CI (95)	
**Patient characteristics**					
Sex (male = ref.)	−0.046	0.000	0.006	−0.058	−0.033
Age (41–64 = ref.)					
• 0–12	−0.699	0.000	0.020	−0.740	−0.660
• 13–17	−0.043	0.013	0.017	−0.077	−0.009
• 18–40	0.314	0.000	0.009	0.296	0.332
• ≥65	−0.618	0.000	0.008	−0.634	−0.601
Diagnosis (not reimbursed = ref.)	−2.993	0.000	0.013	−3.018	−2.969
Number of times physiotherapy^B^	0.217	0.000	0.011	0.196	0.238
0–12*reimbursed	2.170	0.000	0.030	2.111	2.229
13–17*reimbursed	1.098	0.000	0.038	1.023	1.173
18–40*reimbursed	0.397	0.000	0.020	0.359	0.436
≥65*reimbursed	0.076	0.000	0.019	0.039	0.112
**Practice characteristics**					
How urban (highly = ref.)^C^					
• Moderate	0.066	0.588	0.122	−0.173	0.305
• Rural	0.229	0.026	0.103	0.027	0.432
Average age of patients in the practice	−0.002	0.645	0.005	−0.013	0.008
Average percentage of diagnosis for which treatments are reimbursed within a practice^D^	−0.792	0.019	0.339	−1.456	−0.129
Number of therapists per practice	0.012	0.000	0.003	0.006	0.018
Dominant health insurer in the area of the practice (A = ref.)					
• B	0.130	0.303	0.125	−0.117	0.375
• C	−0.006	0.991	0.419	−0.826	0.817
• D	−0.501	0.421	0.622	−1.721	0.719
• E	0.193	0.194	0.149	−0.098	0.485
• F	0.111	0.482	0.158	−0.199	0.422
• G	0.051	0.836	0.247	−0.433	0.535
Intercept	1.211	0.000	0.320	0.583	1.839
Variation practice level^E^	1.110		0.069	0.983	1.253

Data source: Nivel Primary Care Database (2022).

*p < 0.05; **p < 0.01; ***p < 0.001.

*N* patients = 776,882 ; *N* practices = 593.

Notes:

A ) Physiotherapy = 98,09%; exercise therapy = 1,91%.

B ) Number of times physiotherapy = the number of times a patient went to visit the physiotherapist in 2022.

C ) The level of urbanization is determined by the average ambient address density, categorized as follows: (1) Highly urbanized area: with an average ambient address density of 1,500 or more addresses per square kilometre. (2) Moderately urbanized area: characterized by an average ambient address density ranging from 1,000 to 1,500 addresses per square kilometre. (3) Less urban/rural area: exhibiting an average ambient address density of 999 or fewer addresses per square kilometre (39).

D ) Average percentage of diagnosis for which treatments are reimbursed within a practice: we calculated the average diagnosis by assessing the percentage of reimbursed diagnoses within a practice.

E ) ICC = 1.110/(1.110+3.29) = 0.252; ICC = intraclass correlation coefficients.

The practice factors associated with the use of direct access to physiotherapy were: the number of therapists per practice, the average rate reimbursed diagnoses of patients in a practice, and whether the practice is in a rural or highly urban area. The more therapists working in a practice, the more people use direct access. As the percentage of the category of reimbursed diagnoses within a practice increased, the likelihood of the use of direct access decreased. Furthermore, within practices in rural areas the likelihood of using direct access increased compared to those in highly urban areas.

#### Speech therapy

The patient characteristics associated with direct access to speech therapy were: age, SES, and diagnosis (Table 5). Across all age groups, except those aged 65 years or older, the likelihood of accessing speech therapy via direct access was higher compared to those aged 41–64. The likelihood was highest for individuals aged 18–40. There was no significant difference in the use of direct access between those aged 65 or older and those aged 41–64. A higher SES based on postal codes was associated with a higher likelihood of using direct access. Additionally, individuals with an articulation disorder were more likely to use direct access compared to those with a language disorder. There were no differences in the use of direct access for other diagnoses (sensory motor skills disorders and the category other disorders) compared to language disorders. Additionally, the association between diagnosis categories and direct access varied across age groups.

**Table 5.** Speech therapy: patient and practice characteristics associated with the use of direct access

	Coef	*P* value	SE	CI (95)	
**Patient characteristics**					
Sex (male = ref.)	0.060	0.139	0.040	−0.019	0.140
Age (41–64 = ref.)					
• 0–12	2.403	0.001	0.737	0.959	3.847
• 13–17	2.346	0.002	0.772	0.833	3.858
• 18–40	3.452	0.000	0.903	1.682	5.222
• ≥65	0.043	0.959	0.836	−1.595	1.681
SES^A^	0.309	0.002	0.099	0.115	0.504
Diagnosis (language disorder = ref.)					
• Articulation disorder	2.103	0.007	0.778	0.579	3.627
• Abnormality sensory motor skills mouth	1.007	0.185	0.761	−0.483	2.498
• Other^B^	1.134	0.127	0.743	−0.323	2.591
0–12*articulation disorder	−1.930	0.013	0.780	−3.458	−0.402
0–12*sensory motor skills mouth	−1.889	0.013	0.764	−3.385	−0.393
0–12*other	−0.802	0.283	0.747	−2.266	0.663
13–17*articulation disorder	−1.549	0.077	0.875	−3.264	0.167
13–17*sensory motor skills mouth	−2.971	0.000	0.809	−4.557	−1.385
13–17*other	−1.414	0.081	0.811	−3.004	0.177
18–40*articulation disorder	−2.400	0.013	0.970	−4.300	−0.500
18–40*sensory motor skills mouth	−2.193	0.019	0.934	−4.024	−0.363
18–40*other	−2.710	0.003	0.915	−4.503	−0.916
≥65*articulation disorder	−1.736	0.059	0.918	−3.536	0.063
≥65*sensory motor skills mouth	−0.141	0.873	0.878	−1.861	1.580
≥65*other	−0.285	0.739	0.855	−1.961	1.390
**Practice characteristics**					
How urban (highly = ref.)^C^					
• Moderate	0.233	0.613	0.462	−0.672	1.138
• Rural	−0.134	0.767	0.451	−1.017	0.750
Average age of patients in the practice	−0.001	0.993	0.087	−0.171	0.172
Average SES of patients in the practice^A^	1.023	0.432	1.302	−1.530	3.357
Number of therapists per practice	−0.049	0.090	0.029	−0.106	0.008
Dominant health insurer in the area of the practice (A = ref.)					
• B	0.721	0.150	0.502	−0.262	1.704
• D	−2.755	0.149	1.909	−6.497	0.987
• E	1.670	0.003	0.561	0.571	2.769
• F	−0.117	0.819	0.512	−1.120	0.886
• G	−0.293	0.720	0.816	−1.893	1.308
Intercept	−3.592	0.000	1.016	−5.583	−1.601
Variation practice level^D^	2.729		0.409	2.034	3.661

Data source: Nivel Primary Care Database (2022).

*N* patients = 21,201; *N* practices = 110.

Notes:

A ) SES= socioeconomic status, which is based on affluence, educational attainment, and labour market participation, is publicly available and reflects the average SES of the neighbourhood (32).

B ) Other included hearing impairment, voice impairment, reading and writing impairment, and COVID-19 infection.

C ) The level of urbanization is determined by the average ambient address density, categorized as follows: (1) Highly urbanized area: with an average ambient address density of 1,500 or more addresses per square kilometre. (2) Moderately urbanized area: characterized by an average ambient address density ranging from 1,000 to 1,500 addresses per square kilometre. (3) Less urban/rural area: exhibiting an average ambient address density of 999 or fewer addresses per square kilometre (39).

D ) ICC = 2.729/(2.729 + 3.29) = 0.453; ICC = intraclass correlation coefficients.

Only one practice effect was found, related to the dominant health insurer in the area. When health insurer E was the dominant health insurer in the area of the practice, the likelihood of using direct access was higher compared to an area where health insurer A was the dominant health insurer.

#### Dietitians

The patient characteristics associated with direct access to dietitians were: sex, age, SES, and diagnosis (Table 6). Women were more likely to use direct access to dietitians than men. Children aged 0–12 and adults aged 18–40 were more likely to use direct access than those aged 41–64. The likelihood was the same for children aged 13–17 as for people aged 41–64. Individuals aged 65 or older were less likely to use direct access compared to those aged 41–64. A higher SES, derived from postal codes, was associated with an increased use of direct access. Diagnosis was also associated with the use of direct access. Patients defined as being overweight or those categorized as ‘other’ were more likely to use direct access compared to those with medical conditions. Also the effect of diagnosis varied across age groups.

**Table 6.** Dietitian: patient and practice characteristics associated with the use of direct access

	Coef	*P* value	SE	CI (95)	
**Patient characteristics**					
Sex (male = ref.)	0.364	0.000	0.055	0.257	0.472
Age (41–64 = ref.)					
• 0–12	0.936	0.000	0.264	0.419	1.453
• 13–17	0.514	0.068	0.282	−0.039	1.067
• 18–40	0.519	0.000	0.100	0.323	0.716
• ≥65	−0.393	0.000	0.096	−0.581	−0.206
SES^A^	0.355	0.009	0.136	0.089	0.622
Diagnosis (medical = ref.)					
• Overweight	1.237	0.000	0.091	1.059	1.414
• Other^B^	0.940	0.000	0.129	0.688	1.193
0–12*Overweight	−1.045	0.001	0.312	−1.656	−0.434
0–12*Other	−1.311	0.000	0.312	−1.922	−0.700
13–17*Overweight	0.097	0.782	0.351	−0.590	0.783
13–17*Other	−0.609	0.097	0.367	−1.329	0.111
18–40*Overweight	−0.531	0.000	0.141	−0.807	−0.254
18–40*Other	−0.374	0.046	0.187	−0.741	−0.007
≥65*Overweight	−0.209	0.207	0.166	−0.534	0.116
≥65*Other	−1.119	0.000	0.170	−1.452	−0.786
**Practice characteristics**					
How urban (highly = ref.)^C^					
• Moderate	−0.156	0.715	0.666	−1.462	1.150
• Rural	−0.227	0.618	0.591	−1.385	0.932
Average age of patients in the practice	−0.085	0.005	0.030	−0.144	−0.026
Average SES of patients in the practice^A^	0.080	0.975	2.497	−4.814	4.973
Number of therapists per practice	−0.287	0.000	0.076	−0.435	−0.139
≥50% medical conditions (<50% = ref.)^D^	−0.489	0.395	0.574	−1.614	0.637
Dominant health insurer in the area of the practice (A = ref.)					
• B	1.221	0.104	0.752	−1.520	2.696
• C	1.824	0.246	1.573	−1.259	4.907
• E	1.095	0.135	0.733	−0.342	2.532
• F	1.622	0.036	0.775	0.104	3.142
• G	−0.288	0.885	1.997	−4.203	3.626
Intercept	2.297	0.140	1.555	−0.751	5.345
Variation practice level^E^	2.984		0.588	2.029	4.390

Data source: Nivel Primary Care Database (2022).

*N* patients = 15,470; *N* practices = 62.

A ) SES= socioeconomic status, which is based on affluence, educational attainment, and labour market participation, is publicly available and reflects the average SES of the neighbourhood (32).

B ) Other encompassed disorders related to nutrition such as food allergies or food intolerance, general symptoms such as swallowing complaints or chewing and dental problems, and artificial nutrients.

C ) The level of urbanization is determined by the average ambient address density, categorized as follows: (1) Highly urbanized area: with an average ambient address density of 1,500 or more addresses per square kilometre. (2) Moderately urbanized area: characterized by an average ambient address density ranging from 1,000 to 1,500 addresses per square kilometre. (3) Less urban/rural area: exhibiting an average ambient address density of 999 or fewer addresses per square kilometre (39).

D ) Mean diagnosis per practice: we classified practices based on their treatment of medical conditions: those that treated medical conditions in 50% or more of cases were categorized as primarily medical, while those with less than 50% were classified as non-medical.

E ) ICC = 2.984/(2.984 + 3.29) = 0.476; ICC = intraclass correlation coefficients.

Practice characteristics associated with direct access to dietitians included the average age of the patients per practice, the number of therapists working in the practice, and the dominant health insurer in the area where the practice is located. As the average age of patients in a practice or the number of therapists increased, the use of direct access decreased. Additionally, when health insurer F was the dominant health insurer in the area, the percentage of direct access usage was higher compared to areas where health insurer A was the dominant health insurer.

#### Differences between practices

In general, we observed relatively large differences between practices. The intraclass correlation coefficients revealed that the largest differences at the practice level were seen among dietitians. The proportion of the variance explained by practice differences was 47,6%. Speech therapy showed a similar trend, with 45.3% of the variance explained by practice differences. In physiotherapy, practice differences accounted for 25.2% of the variance.

## Discussion

This study identified factors associated with the use of direct access for physiotherapy, speech therapy, and dietitian services using electronic health records. Across all disciplines, patients who use direct access are, in general, younger and have a higher SES. Moreover, we found that whether a diagnosis of the patient is reimbursed, and the number of times that someone has been to the physiotherapist in the same year, are associated with the use of direct access within physiotherapy. For physiotherapy, men used direct access more often, while for dietitian services, women did. These patterns of association, and the direction of the relationships, align with previous research on physiotherapy (Leemrijse *et al.*, 2008, Scheele *et al.*, 2014, Babatunde *et al.*, 2020), our study extends this knowledge to dietetics and speech therapy.

The lower use of direct access by people aged 65 or older could be due to them having a higher incidence of chronic symptoms and comorbidities (Fabbri *et al.*, 2015, Organisation for Economic Co-operation and Development, 2021a). This entails the chance of referrals. Moreover, the probability of an existing relationship with their GP is higher, making it more likely that patients will consult their GP first. Additionally, older people may be less aware of direct access. The increased likelihood of using direct access, with more frequent visits to the physiotherapist within a year, suggests that some patients may not have been initially aware of this option, or were unsure which HCP to contact for their health concerns, leading them to visit a GP first.

The lower use of direct access among patients with lower SES may be attributed to limited health literacy, which is a known risk factor associated with this group (Lee *et al.*, 2010, van der Heide *et al.*, 2013, Sun *et al.*, 2013, Stormacq *et al.*, 2019). Patients with limited health literacy may lack complete information, or even the ability to anticipate their healthcare needs and, therefore, to make well-informed decisions about healthcare providers (Nutbeam and Kickbusch, 1998). Therefore, individuals with limited health literacy may be less aware of direct access options or may have greater difficulty selecting the appropriate healthcare provider. As a result they may go to their GP for advice more often compared to those with higher health literacy. An economic explanation could be that a consultation with a GP in the Netherlands is reimbursed, whereas consultations with allied health professionals often require an out-of-pocket payment or are only reimbursed for individuals with supplementary insurance. Finally, patients with lower SES and/or limited health literacy are more likely to suffer from multimorbidity (Pathirana and Jackson, 2018), increasing the chance of visiting physicians and therefore of referrals.

We did not find previous research that included practice characteristics associated with direct access and therefore cannot compare our results to other literature. We found considerable variation between practices, but few practice-level characteristics were significantly associated with direct access use. One factor that may influence this is the dominant health insurer in a practice’s area. In areas where speech therapy or dietitian practices made no use of direct access, the dominant health insurer in those areas was more often Health Insurer A compared to the overall sample (approximately 35% vs. 18%). This may indicate that insurer policies set by dominant insurers influence practice behaviours, even when patients are insured by different providers. Further investigation is needed to understand better what impact insurer policies have on the use of direct access at the practice level.

The variation between practices may also be influenced by factors not captured in this dataset. As the Nivel PCD is limited to formally registered data, more nuanced contextual elements are not included. These may include agreements between practices and GPs that affect referral patterns, internal policies on the use of direct access, or a focus on specific conditions that typically require a referral (Vektis, 2024b, Kennis- en exploitatiecentrum Officiële Overheidspublicaties, 2024). Therapist-level factors may also play a role, such as personal preferences regarding direct access. Additionally, allied health professionals are required to complete specific training before they are permitted to apply direct access in practice. Not all allied health professionals may have completed this training which can lead to differences in its use between practices. We expect these factors to have an impact on the use of direct access, and future research should incorporate them to allow for more accurate comparisons across practices.

The factors explaining differences in the use of direct access between practices are not yet fully understood. However, the significant differences in the use of direct access between practices suggest that policy efforts should also focus on the practice level to enhance its use and improve efficient care.

### Strengths and limitations

The large sample size of the datasets we used, reduces the likelihood of random errors, results in more consistent findings with repeated measures, and increases both internal and external validity. Moreover, to our knowledge, no other studies have investigated associations with direct access across three allied health professions, including practice-level variation.

However, a limitation of the study is the potential for misclassifications of the dependent variable. When a patient directly accesses services, it does not necessarily mean they have not consulted a GP for the same complaint. A GP may advise a patient to visit an allied health professional without issuing a formal referral. In such cases, the patient may be incorrectly recorded as having come through direct access and our results may be an underestimation. The extent of this cannot be determined from our current dataset. GP data would be required to assess this more accurately.

Another limitation is that we had to remove practices with an average of 0% direct access. This accounted for almost one-fourth of all dietitian and speech therapy practices. These had to be excluded from the dataset. Although the remaining sample size was still substantial, it is unfortunate because the practices we excluded are particularly interesting for understanding if they differ fundamentally from others. While we compared the populations of these practices, we were unable to conduct a full analysis that included them.

### Future research

One recommendation for future research is to investigate further the effect of diagnosis on direct access, rather than including a rough classification based on diagnoses. One could specifically identify diagnoses such as knee injuries for which GPs expect patients to access a physiotherapist directly. By comparing patients who use direct access with those who are referred for these specific diagnoses, researchers can analyse whether these groups differ significantly from one another. Another recommendation is to examine how the patient and practice characteristics associated with direct access have evolved over the years. This longitudinal analysis will provide insights into how these associations have changed over time and help identify which patient groups should be the focus of future policies.

To better understand the variation between practices and the factors driving these differences, engaging with allied health professionals to explore their perspectives on direct access may yield valuable insights. In addition, health insurers and healthcare policies appear to influence the use of direct access. Further research should examine their role in more detail to gain a clearer understanding of their impact.

## Conclusion

There are significant associations between patient and practice characteristics and the use of direct access to allied health professionals in primary care. To increase the use of direct access and improve the efficiency of healthcare, policies could target specific patient groups, such as those with lower SES. However, it appears that substantial differences in the use of direct access emerge at the practice level. For policies to address this effectively, a deeper understanding of the factors driving these variations at the practice level is essential.
